# Computer assisted Doppler waveform analysis and ultrasound derived turbulence intensity ratios can predict early hyperplasia development in newly created vascular access fistula: Pilot study, methodology and analysis

**DOI:** 10.1177/20480040211000185

**Published:** 2021-03-20

**Authors:** Matthew Bartlett, Vanessa Diaz-Zuccarini, Janice Tsui

**Affiliations:** 1 Department of Surgery & Interventional Medicine, University College London, London, UK; 2 Royal Free London NHS Foundation Trust, London, UK; 3 Department of Mechanical Engineering, University College London, London, UK

**Keywords:** Cardiovascular imaging agents/techniques, Diagnostic Testing, ‘Cardiology, Other diagnostic testing’, Diagnostic Testing, ‘Cardiology, Smooth muscle proliferation and differentiation’, Vascular biology, Cardiology

## Abstract

**Objectives:**

Following surgical creation of arterio-venous fistulae (AVF), the desired outward remodeling is often accompanied by the development of neointimal hyperplasia (NIH), which can stymie maturation and may lead to thrombosis and access failure. The aim of this study was to investigate the feasibility of using a non-invasive test, to detect and quantify the turbulent flow patterns believed to be associated with NIH development.

**Design:**

This was a prospective, observational study. Ultrasound derived turbulence intensity ratios (USTIR) were calculated from spectral Doppler waveforms, recorded from newly formed AVF, and were compared with haemodynamic and structural changes observed during the initial maturation period.

**Setting:**

Measurements were obtained by accredited Clinical Vascular Scientists, at the Royal Free Hospital, London.

**Participants:**

Patients with newly created AVF were invited to participate in the study. A total of 30 patients were initially recruited with 19 participants completing the 10 week study protocol.

**Outcome measures:**

The primary outcome measure was the development of NIH resulting in a haemodynamically significant lesion.

The secondary outcome was successful maturation of the AVF at 10 weeks.

**Results:**

Elevated USTIR in the efferent vein 2 weeks post surgery corresponded to the development of NIH formation (P = 0.02). A cut off of 6.39% predicted NIH development with a sensitivity of 87.5% and a specificity of 80%.

**Conclusion:**

Analysis of Doppler waveforms can successfully identify deleterious flow patterns and predict inward luminal remodelling in maturing AVF. We propose a longitudinal follow up study to assess the viability of this technique as a surveillance tool.

## Introduction

Current recommendations for arterio-venous fistula (AVF) surveillance are focused on clinical assessment and flow volume measurements,^1^ however this approach will often only identify problems, which have already become established. The development of neointimal hyperplasia (NIH) in the efferent vein is the primary cause of AVF failure,^2^ and a surveillance tool which enabled us to identify those at highest risk would have the potential to improve long term patency rates.

It has long been recognised that turbulent flow patterns associated with end-side anastomosis configurations can lead to NIH development, triggered by endothelial injury and a subsequent proliferation of myofibroblasts in the extracellular matrix.^3^ Oscillatory shear index (OSI) quantifies the deviation of wall shear stress (WSS) from the direction of the net flow field,^4^ and has been shown to correlate with regions of localised NIH development.^5,6^ OSI can be calculated using computational fluid dynamics (CFD) modelling, but the time and computational expense associated with this technique is prohibitive to use as a routine clinical tool. The uniplanar nature of ultrasound imaging, combined with the inability to insonate the entire volume of the lumen in long section, makes it impossible to fully assess complex 3-dimensional flow fields, meaning that OSI cannot be calculated during routine imaging. We know that OSI values tend to be higher in regions of turbulence, the aim of this pilot study was to investigate the feasibility of using non-invasive Doppler ultrasound, combined with computer assisted waveform analysis to quantify turbulent flow, and provide a simplified method for identifying patients, with newly created AVF, who may be at increased risk of negative luminal remodeling.

## Methods

### Ethics

Following ethical approval, ESRF patients, over the age of 18, undergoing autogenous AVF formation surgery were approached and consented to participate in the study (n = 30). Aside from the additional ultrasound scans, participation in the study did not change the treatment pathway of these patients.

We have no conflicts of interest to declare.

### Scanning protocol

Each patient was invited to attend 2 ultrasound scans in addition to their routine care; 1 pre maturation scan at 2–4 weeks post surgery, and a second post maturation at 6–10 weeks.These time frames were based on NKF-KDOQI guidelines, which recommend a maturation time of 4–6 weeks prior to cannulation.^1^ All patients received an assessment 10 weeks post surgery (±6 days) as part of their routine care.

Digital audio recordings were made of the Doppler shifted spectrum, from the feeding artery, the juxta-anastomotic region and the efferent vein. Arterial measurements were obtained in a straight, uniform segment of the artery at least 10 cm above the anastomosis, juxta-anastomotic measurements were taken in the vein 1 cm from the anastomosis, and proximal venous measurements were recorded 4–7 cm above the anastomosis; an in-vitro study by Sivanesan et al. found that the relative turbulence intensity was comparably uniform throughout this segment.^7^

The Doppler sample volume used to collect the audio data was opened to encompass the entire width of the vessel lumen, and the beam vessel angle was maintained at 60**°**. We acknowledge that the large sample volume may result in reduced spatial resolution, but felt that maximizing measurement repeatability was vital if this technique was to see successful translation into a clinical setting.

### Equipment

Scans were performed on a Toshiba Aplio 500 ultrasound system, which was modified to allow for the audio signal to be routed to an external analogue-digital convertor. Doppler information was obtained using a 4 MHz transmit frequency, and audio was recorded using a sample rate of 44.1 kHz. A piezo contact microphone was coupled to a fingertip pulse oximeter, to simultaneously capture gating pulses, in the form of audio chirps recorded to a separate channel of the recording software. The gate signals were recorded from the contralateral arm to avoid loss of sensitivity in patients experiencing steal syndrome; a phenomenon, which is widely documented in patients with AVF.^8^ Analysis of the Doppler audio was performed retrospectively, after the week 10 assessments, ensuring that at the time of the scans the reporting scientist was blinded to the findings.

### Population

For the purpose of this feasibility study, a small cohort of 30 patients were recruited (Male = 13, Female = 17), 6 suffered early thrombosis, excluding them from further involvement, 2 patients never underwent AVF surgery, 1 patient withdrew, 1 patient was unable to attend due to hospital admission, and 1 patient was lost to follow up. This reduced our study population to 19 patients, summarised in Table 1.

**Table 1.** Study population.

	n	Radio-cephalic	Brachio-cephalic	Brachio-basilic	Attended both scans	Attended pre-maturation scan	Attended post-maturation scan
Male	8	5	1	2	6	8	6
Female	11	5	5	1	6	10	7
Total	19	10	6	3	12	18	13

Of the patients who suffered from early thrombosis 5 had Radio-cephalic (R–C) AVF and 1 had a brachio-basilic (B–B) AVF. 4 were female and 2 were male.

### Ultrasound turbulence intensity ratio

The Ultrasound Turbulence Intensity Ratio (USTIR) was derived from an offline computational analysis of the Doppler audio spectrum.

Angle corrected Doppler ultrasound measurements use changes in the Doppler shifted ultrasound frequency to monitor flow velocities in real time. These measurements work on the assumption that laminar flow is maintained throughout the cardiac cycle, with the direction of flow remaining parallel to the vessel walls. We know that in the presence of disturbed or turbulent flow these assumptions are not true, meaning that the frequencies present in the Doppler shifted spectrum are representative of not just changes in the velocity, but also fluctuations in flow direction.

The changes in the Doppler shifted frequencies, due to variations in both flow velocity and direction, can be divided into those which are cyclical, occurring due to cardiac output and vessel compliance, and those which are random, and are not repeated with each cardiac cycle. These random fluctuations in the frequency spectrum can be assumed to represent the turbulent component of the flow field.

**Figure 1.**
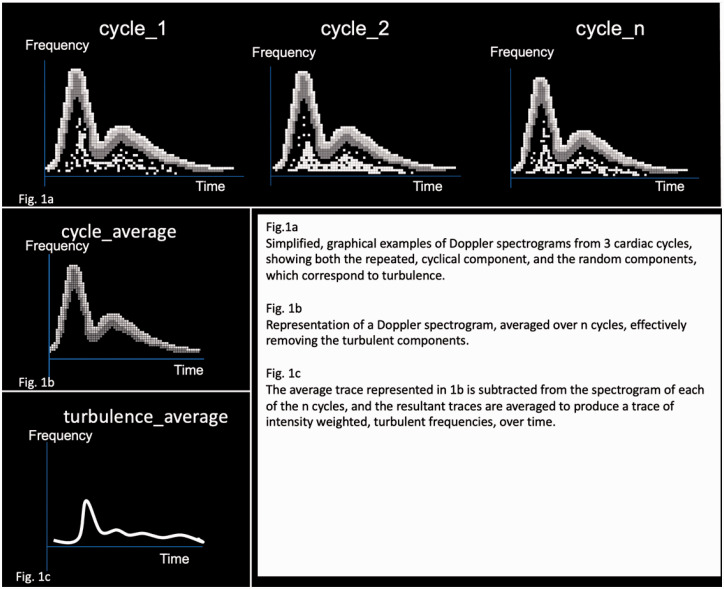
Isolation of turbulent flow components based on a simple ensemble averaging technique.

An ensemble averaging technique was employed to remove the cyclical components from the Doppler shifted signals, isolating the portion of the audio spectrum relating to random fluctuations and turbulence, a simplified graphical representation of this process is shown in Figure 1.

Matlab *(Natick, Massachusetts: The MathWorks Inc)* was used to detect the gate pulses and slice the audio files into 25 individual cardiac cycles, before carrying out Fourier transforms generating a spectrogram for each cardiac cycle. The average, intensity weighted frequency shifts were extracted and used to calculate the mean flow velocity ($\bar{u}$).

Turbulence intensity (TI) is equal to the root mean squared deviation about the average velocity.^9^

(1) $$TI=\sqrt{\frac{\sum {\left(u-\bar{u}\right)}^{2}}{n}}$$

Instantaneous turbulence intensity (TI)

TI gives us a value for the average velocity of the turbulent components of the flow field and is usually expressed in cm/s. Given the large variance in AVF flow velocities observed between patients, and the limitations of measuring non-axial flow velocities with ultrasound, we normalized this value by the mean velocity ($\bar{u}$) to obtain the Ultrasound Turbulence Intensity Ratio (USTIR).

(2) $$\mathrm{USTIR}=\;\frac{TI}{\bar{u}}\times 100$$

Ultrasound Turbulence Intensity Ratio

USTIR gives us an estimated value for the percentage of the total flow field exhibiting turbulent behavior. This provides a method of quantifying turbulence, which is comparable between patients and independent of the changes in $\bar{u}$ observed throughout the maturation process.

Viability of the ensemble averaging method for calculating TI in the presence of cardiac variability has been validated in vitro.^10^ We compared the pulse-oximeter output with the Doppler audio signals using Sonic Visualiser software (QMU, London), demonstrating reliable gating even in the presence of irregular cardiac output. There was an average delay of 430 ms between the onset of systolic acceleration and the gate pulse. For the purpose of this proof of concept study, timing was manually corrected for each patient, but it is worth noting that using a generic time correction of 400 ms for all patients resulted in negligible differences from the results presented below. This is consistent with previous studies which found that TI is greatest during systolic deceleration and early diastole,^11,12^ so clipping of the initial systolic acceleration due to imperfections in timing correction does not impact the overall USTIR values.

## Results

Patients were grouped according to findings on their 10 week assessment, with the primary outcome being the presence of significant NIH within the efferent vein, and the secondary outcome being maturation of the AVF; based on the definition proposed in the NKF-KDOQI guidelines, which stipulate flow volumes greater than 600 ml/min, vein diameter greater than 6 mm and vein depth of less than 6 mm.^1^ Significant NIH was defined as any region where NIH caused a reduction in the lumen of the efferent vein, resulting in localised haemodynamic changes detectable on Doppler ultrasound. Outcomes are summarised in Table 2.

**Table 2.** Outcomes at 10 weeks.

	Significant NIH		Maturation at week 10	
	Yes	No	Yes	No
Total	8	11	7	12
Male	4	4	3	5
Female	4	7	4	7
Radio-Cephalic	5	5	3	7
Brachio-Cephalic	2	4	2	4
Brachio-Basilic	1	2	2	1

Levene’s test was used to confirm homogeneity of variance between the groups and a 2 tailed independent T-test was used to assess for statistical significance of the differences in USTIR. Average values of USTIR are documented in Table 3.

**Table 3.** Average USTIR values.

	Significant NIH		No NIH		Successful Maturation		Unsuccessful Maturation	
	Average USTIR	Standard Deviation	Average USTIR	Standard Deviation	Average USTIR	Standard Deviation	Average USTIR	Standard Deviation
Scan 1 Vein	8.87%	3.64	5.57%	1.64	6.28%	1.92	7.52%	3.70
Scan 1 J-A	7.79%	2.05	6.96%	3.92	6.26%	2.69	8.01%	3.38
Scan 1 Artery	8.41%	3.78	6.01%	2.79	5.35%	1.21	8.18%	3.91
Scan 2 Vein	7.11%	1.96	7.70%	4.37	6.45%	0.93	7.83%	3.98
Scan 2 J-A	11.15%	7.60	8.24%	2.26	12.30%	7.98	8.38%	3.76
Scan 2 Artery	8.23%	6.88	4.89%	0.65	4.97%	0.75	7.11%	5.72

Average USTIR_vein_ was higher in R-C AVF (8.1% vs 6.0% in B-C AVF and 5.1% in B-B AVF), however this was not statistically significant (P = 0.24). There was no significant difference between USTIR_vein_ measurements in male and female patients, (7.5% vs 6.6%: P = 0.55), and after excluding the patients who suffered early post surgical thrombosis, neither gender nor AVF location were a significant factor in AVF outcomes.

### Neointimal hyperplasia development

**Figure 2.**
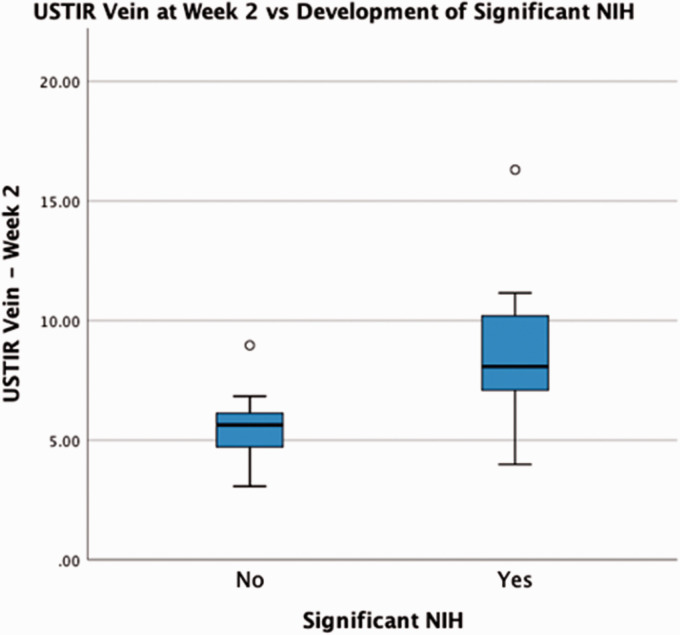
USTIR_(vein)_ on the pre-maturation scan vs Presence of significant NIH at week 10.

Significant NIH development within the efferent vein was identified in 42% of the study population (n = 8). On the pre-maturation scans (2–4 weeks post surgery) a significantly higher USTIR was recorded from the efferent vein (USTIR_vein_) in this group of patients; an average of 8.87%, compared to 5.57% in the patients who did not develop significant NIH (P = 0.02) as shown in Figure 2. A ROC curve analysis was performed, demonstrating that within our study cohort, USTIR_vein_ values >6.39% on a pre-maturation assessment, predicted NIH development with a sensitivity of 87.5% and a specificity of 80% (P = 0.016).

Measurements of USTIR_vein_ on the post maturation scan (6–10 weeks) showed almost no difference between the 2 groups. The distribution of values was much greater than those on the pre-maturation scans, with a large increase in standard deviation observed amongst the patients, whose AVF did not exhibit significant NIH formation.

USTIR in the juxta-anastomotic vein (USTIR_JA_) showed correlation with NIH development, with average values of 11.15% vs 8.24% on post maturation scan, however this trend was not found to be statistically significant (P = 0.35).

USTIR in the artery (USTIR_artery_) also showed correlation with NIH development, with average values of 8.41% vs 6.01%, and 8.23% vs 4.89% on the pre and post-maturation scans respectively, these correlations did not reach statistical significance (P = 0.14 and p = 0.22). The 3 patients with the highest USTIR_artery_ measurements, all had significant inflow (arterial) disease reported on their pre-maturation ultrasound scans.

### AVF maturation at 10 weeks

**Figure 3.**
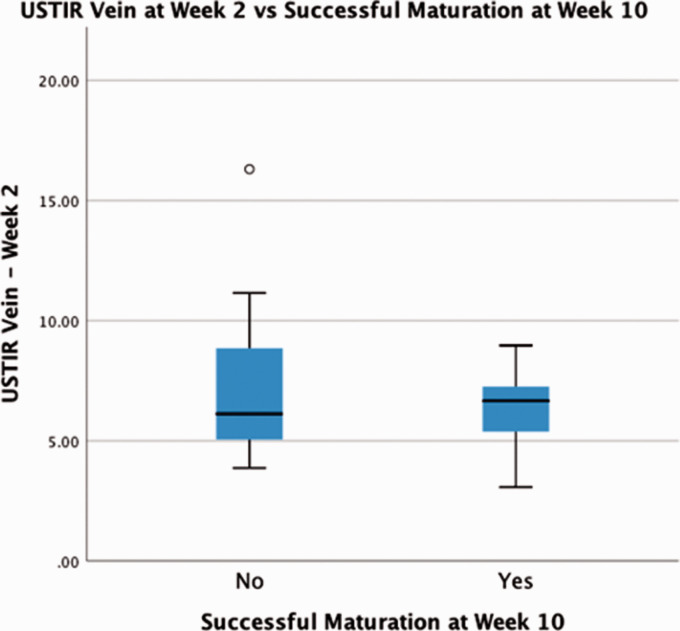
USTIR_(Vein)_ on the pre-maturation scan vs Successful maturation at week 10.

Despite being a strong predictor of NIH development, pre-maturation USTIR_vein_ ability to predict successful maturation at 10 weeks was not statistically significant. Patients whose AVF failed to mature had an average USTIR_vein_ of 7.52%, compared to 6.26% in those who met the maturation criteria (P = 0.43) as shown in Figure 3.

## Discussion

We have demonstrated a novel, non-invasive Doppler ultrasound technique, for detecting and quantifying flow disturbances within newly created AVF. In-vivo USTIR measurements were obtained using an ensemble averaging technique, with cardiac gating signals acquired from a simple fingertip pulse oximeter, removing the requirement for an ultrasound system equipped with ECG capabilities.

Pre-maturation measurements of USTIR in the efferent vein were a strong predictor of NIH development (P = 0.02). The range of values for USTIR_vein_ was lower than in-vitro measurements reported in the literature, obtained using a laser Doppler anemometry technique.^7^ This discrepancy may be partly due to approximations made by in-vitro flow circuits, but the major factor is likely to be the size and positioning of the sample volume used for collection of our Doppler data. As discussed in the methods we utilised a large sample volume, incorporating the entire luminal cross-section, however the dimensions of the sample volume in the slice thickness plane (perpendicular to the image plane) is fixed on most ultrasound systems, and is dictated by the geometry of the ultrasound beam. Working on the assumption that turbulence in this section of the vein is less prevalent in the centre of the vessel, we can hypothesise that absence of Doppler information from the lateral walls of the vessel, combined with the large sample volume length in the image plane, result in an underestimation of the true TIR. It is for this reason that we refer to Ultrasound derived Turbulence Intensity Ratio (USTIR) throughout this paper; in order to differentiate these measurements from turbulence intensity index values obtained using more conventional methods.

Despite the strong correlation between USTIR_vein_ on the pre-maturation scans and NIH development, the same measurements obtained on the post-maturation scans were not statistically significant in predicting NIH. This study was designed primarily to investigate the viability of detecting potentially detrimental flow disturbances in newly formed AVF, and the follow up period may not have been of a sufficient duration to identify the impact of USTIR remaining elevated, or continuing to increase beyond the initial maturation period.

A correlation was identified between those patients who exhibited an increase in USTIR_vein_ over the course of the study and those whose AVF failed to meet the maturation criteria, however statistical significance could not be demonstrated from this dataset. We can hypothesise that the low rate of maturation (37%) may indicate that the criteria used were too stringent. A more robust approach may have been to assess how many of the patients underwent successful HD via their AVF, however at the time of writing a number of the patients recruited for the study remain in pre-dialysis ESRF, meaning that this metric could not be utilised.

The requirement to keep the Vascular Scientists performing the ultrasound scans blinded to the USTIR measurements meant that the opportunity to extend the follow up duration, by collecting further data within a meaningful timeframe had been missed by the time data analysis was completed. The discrepancy between the significance of USTIR_vein_ at the 2 time points highlights the need for further longitudinal studies to verify the efficacy of this technique as a monitoring tool for AVF.

The highest recorded values of USTIR_artery_ came from arterial segments, with pre-existing atherosclerotic changes. It is possible that the increased prevalence of turbulent flow stymied maturation of these vessels resulting in further elevations of USTIR. We must also consider the fact that any pre-existing focal stenotic lesions will have resulted in increased flow velocities with a corresponding rise in Reynold’s number, predisposing these vessels to disturbed or turbulent flow. It is therefore not possible for us to differentiate between the cause and effect of elevated USTIR_artery_ and the corresponding arterial lesions. We know that pre-existing arterial disease results in poorer vascular access outcomes,^13^ so the relevance of USTIR_artery_ may warrant further investigation with a cohort of patients stratified according to pre-surgical levels of arterial disease.

Lower USTIR_JA_ at 6 weeks corresponded to a decreased incidence of successful maturation, however significant outliers were identified in both groups, and no reliable cut-off for predicting maturation could be extrapolated from the current data set. The haemodynamics in this region of the anatomy are complex and are impacted by a number of variables including the size, shape and angle of the anastomosis.^14^ The presence of reverse arterial flow distal to the anastomosis has also been shown to have a major impact on TI.^7^ We know that some degree of turbulence is inherent at the anastomosis, due to the inertial forces associated with the abrupt change in flow direction. It is likely that the reduced USTIR observed in this region in the failing AVF, is secondary to a reduction in flow volumes and corresponding velocities, resulting from flow limitations elsewhere in the circuit. Further to this CFD studies have shown that the non-restoration of haemodynamic homeostasis at the anastomosis is observed in fully functioning AVF,^15^ suggesting that flow disturbances in this region may not provide a reliable indicator of outcomes.

## Conclusion

USTIR can be reliably measured in-vivo using an ensemble averaging technique to assess Doppler ultrasound recordings from surgically created AVF. Our initial hypothesis that these measurements could be used to detect potentially detrimental shear forces with high directional variability is supported by the strong correlation between elevated USTIR in the efferent vein and the development of NIH resulting in haemodynamically significant lesions. The uni-planar nature of Doppler ultrasound assessment means that the exact nature of these haemodynamic forces cannot be directly assessed using this method, but comparison with patient specific computational fluid dynamics (CFD) may offer further insight.

Although we demonstrated the ability of this technique to predict NIH development in newly created AVF, this did not prove to be a reliable indicator of short-term outcomes (successful maturation), and data on long term AVF patency rates for this cohort of patients is not currently available. We know that following surgical AVF creation, rapid vascular remodelling is initiated accompanied by an increase in access flow volumes.^16^ Our findings support the idea that some degree of NIH development may represent part of the normal mechano-stimulated remodelling process, as the body adapts to restore mechanical homeostasis.^17^ Further research is needed to assess the impact of elevated USTIR_vein_ beyond the initial maturation stage, and how this may correlate to the late stage complications and recurrent stenotic processes observed in many dialysis patients.

Although ROC curve analysis did enable us to establish a level of USTIR, above which NIH development was more likely we cannot recommend that these values are used for clinical decision making at this time. Our preliminary dataset provides a proof of concept for the measurement technique, and supports the idea that USTIR could be used to stratify patients into high and low risk groups, however further investigation and validation with a larger cohort of patients, across a number of different ultrasound systems is required.

We believe that the validation of this measurement technique presents the opportunity to develop a patient focussed surveillance programme; helping to identify those patients who may be at higher risk of developing outflow stenoses within their vascular access sites, and who may benefit from regular imaging based surveillance in addition to the current clinical and flow volume based assessments.
